# The Current State of Naïve Human Pluripotency

**DOI:** 10.1002/stem.2085

**Published:** 2015-07-14

**Authors:** Benjamin T. Dodsworth, Rowan Flynn, Sally A. Cowley

**Affiliations:** ^1^ Sir William Dunn School of Pathology, University of OxfordSouth Parks RoadOxfordUnited Kingdom

**Keywords:** Naïve, Pluripotency, Ground state, Embryonic stem cells, Differentiation

## Abstract

Naïve or ground state pluripotency is a cellular state in vitro which resembles cells of the preimplantation epiblast in vivo. This state was first observed in mouse embryonic stem cells and is characterized by high rates of proliferation, the ability to differentiate widely, and global hypomethylation. Human pluripotent stem cells (hPSCs) correspond to a later or “primed” stage of embryonic development. The conversion of hPSCs to a naïve state is desirable as their features should facilitate techniques such as gene editing and more efficient differentiation. Here we review protocols which now allow derivation of naïve human pluripotent stem cells by transgene expression or the use of media formulations containing inhibitors and growth factors and correlate this with pathways involved. Maintenance of these ground state cells is possible using a combination of basic fibroblast growth factor and human leukemia inhibitory factor together with dual inhibition of glycogen synthase kinase 3 beta, and mitogen‐activated protein kinase kinase (MEK). Close similarity between the ground state hPSC and the in vivo preimplantation epiblast have been shown both by demonstrating similar upregulation of endogenous retroviruses and correlation of global RNA‐seq data. This suggests that the human naïve state is not an in vitro artifact. Stem Cells *2015;33:3181–3186*

**Significance Statement**

The newly discovered state of ‘naïve’™ human pluripotency is not only an extremely interesting biological phenomenon, but also promises to overcome some of the problems posed by conventional ‘primed’™ human pluripotent stem cells. These problems include variable differentiation capability, difficult single‐cell passaging, and low gene editing efficiency – all bottlenecks for applications such as regenerative medicine. A flurry of recent papers describe new ways of accessing naïve human pluripotency. However, there are important differences between these protocols, making this concise yet comprehensive review a timely necessity to navigate the complexities of this emerging field.

## Introduction

**Figure 1:**
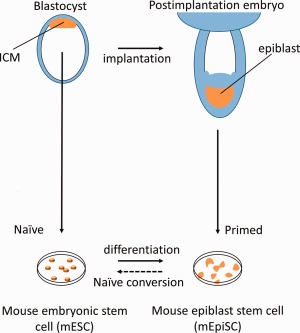
Two states of mouse embryogenesis captured in vitro. mESCs are sourced from the ICM of the preimplantation blastocyst, whereas EpiSCs are in a more differentiated state and are extracted from the post implantation epiblast. Both mESCs and mEpiSCs resemble the state from which they were sourced 8. Abbreviations: ICM, inner cell mass; mESCs, mouse embryonic stem cells; mEpiSCs, mouse epiblast stem cells.

In mice, two pluripotent states have been captured in vitro. Mouse embryonic stem cells (mESCs) are sourced from the inner cell mass (ICM) of the preimplantation blastocyst 1, 2. When derived and maintained using a combination of leukemia inhibitory factor (LIF) and 2i (dual inhibition of extracellular signal‐regulated protein kinases 1/2 [ERK1/2] pathway and glycogen synthase kinase 3 beta [GSK3β]) they are described as being in a naïve or ground state 3. When injected back into an early embryo, these cells can contribute to all lineages without tumorigenesis 4. A more recent discovery has been mouse epiblast stem cells (mEpiSCs—Fig. 1). These are sourced from postimplantation epiblast cells 5, 6 and are termed primed, due to their inability to integrate into a preimplantation blastocyst. They can, however, be differentiated into all three germ layers in vitro. The most striking difference is the very high expression of de novo methyltransferases, which leads to condensing of chromatin 7. Additionally, these cells require basic fibroblast growth factor (bFGF also known as FGF2) and transforming growth factor beta (TGFβ) for self‐renewal, instead of 2i and LIF 3, 5. mEpiSCs can be converted back to the naïve state by transfection with *Klf4* or other reprogramming factors or using small molecules 8, 9.

Naïve pluripotent stem cells have been successfully captured in vitro from primed rhesus monkey induced pluripotent stem cell (iPSC) lines using specialized media containing 2i and LIF 10. Since naïve pluripotent stem cells can be generated from primates, this suggests that the state of naivety might be conserved across species. Using primate cells also allows dissection of genetic background and species to species differences. Primate naïve iPSCs require bFGF, whereas bFGF causes differentiation in mESCs. Additionally, TGFβ is not required for maintenance of primate naïve iPSCs, indicating that TGFβ might not be essential in the human system 10.

Embryogenesis is inherently different between species, which is reflected by the difficulties in generating truly naïve human pluripotent stem cells (hPSCs) in vitro. For ethical reasons, information on human embryogenesis is lacking and many assumptions are made based on the mouse model 11. Despite being sourced from the same point in development as mESCs, hESCs resemble mEpiSCs. Both form large, flat, 2D colonies and require bFGF for self‐renewal. The ability to convert mEpiSCs to mESCs has led to the prediction that naïve hPSCs might also be accessible by reverting primed hESCs. This has prompted several recent publications of strategies to capture the human naïve state, either relying on transgene overexpression 12, 13, 14 or different combinations of small molecule inhibitors 15, 16, 17, 18, 19, 20. Here we review and compare all these published protocols, including a protocol devised by Duggal et al. 16 published in this issue.

## Key Characteristics of the Naïve State

A key difference between naïve and primed cells lie in their differentiation potential. For assessing human cells, Gafni et al. 15 used chimera assays, where human naïve or primed cells are injected into mouse morulas. Unlike the primed cells, the progeny of the naïve cells were subsequently detected in all tissues 15. However, Theunissen et al. 19 found this method unreproducible, since no human cells derived from naïve stem cells were detected when performing the assay in their laboratory, despite using cells generated by Gafni et al. as a control 19. A less rigorous but widely used assay measures teratoma formation following injection of PSCs in immunocompromised mice and assessment of presence of mesoderm, endoderm, and ectoderm lineages. Naïve and primed human pluripotent cells form mature, high grade teratomas 14, 15, 18, 19, 20, with one study suggesting that naïve cells form teratomas of increased volume in a shorter time in comparison to primed cells 17. Naïve PSCs, like primed PSCs, can readily form embryoid bodies containing cells of all three germline lineages 14, 15, 16, 17, 18. Directed differentiation protocols have also been performed 14, 16, 18, 19. Most notably, Duggal et al. 16 show improved efficiency and homogeneity of directed differentiation toward neuronal, mesodermal, and endodermal lineages in comparison to primed cells.

Respiration is different between the two cell types: primed cells are almost entirely glycolytic, whereas metabolism in naïve cells uses greater mitochondrial respiration 14, 20. This shift is also observed in vivo. Before implantation of the blastocyst in mouse, cells rely on oxidative phosphorylation 21, 22, whereas after implantation a shift toward glycolytic metabolism occurs 23. Increasing evidence (reviewed in 24) is emerging that the regulation of energy metabolism is connected with epigenetic modifying machinery, which is also involved in progression from the naïve state.

Naïve cells show higher survival of single cell passaging in comparison to their primed counterparts 15. They also differ in their doubling time of approximately 16 hours instead of 36 in hESC 5. Differences in morphology are also widely reported 14, 15, 16, 17, 19, 20. In their naïve stage, hESCs and mESCs form rounded 3D colonies, whereas primed cells grow in flat monolayers. This may play a role in diffusion of signaling molecules and cell–cell adhesion pathways.

A key observation is the difference in enhancer landscape between naïve and primed cells. Globally, more enhancers are active during the naïve state, whereas inactive enhancer complexes in similar positions are observed in primed cells. These are termed “seed enhancers” and seem to prepare for larger enhancer complexes and reorganization 25, 26. Gafni et al. 15 and Theunissen et al. 19 took advantage of the differential use of enhancers of the *OCT4* gene as a way to assay for optimal naïve maintenance conditions. Although both enhancers activated the gene to the same extent, the proximal enhancer is mainly active in primed cells, whereas the distal enhancer is used in the naïve state 15, 19.

Gene expression is different between naïve and primed cells. These changes have been reported on a global scale 14, 15, 16, 17, 19, 20. Transcript levels have been shown to correlate between naïve hPSC and mESC 14, 19, and this similarity has been used to assess naivety, albeit being cross‐species comparison 19. For a within‐species comparison, Wang et al. analyzed available RNA‐seq data from cells taken from the ICM of early human embryos and compared these expression patterns to naïve cells generated in vitro 27. The authors argue that this comparison to human in vivo data is more relevant than comparisons to mouse, especially since they discovered a primate‐specific transcript, human endogenous retrovirus subfamily H (HERVH), as a key component of naivety. They disrupted either HERVH or its binding partner LBP9 which showed that HERVH is essential for self‐renewal in naïve PSCs 27. Expression of endogenous retroviruses were confirmed by Grow et al. 28, who report high expression of HERVK in preimplantation epiblast cells and in the naïve cell line Elf1 generated by Ware et al. 20 and also in cells converted to the naïve state using the protocol by Chan et al. 17.

RNA methylation has been shown to play a role in the ability to maintain and exit ground state pluripotency. Two recent publications by Batista et al. and Geula et al. 29, 30 came to the same conclusion: Knockout of the N^6^‐methyladenosine (m^6^A) transferase *METTL3* causes reduced m^6^A RNA methylation and failure to resolve the naïve state. These cells have been referred to as being hyperpluripotent due to their inability to differentiate. Transcripts marked with m^6^A decay faster and therefore allow the cells to make changes and differentiate 29, 30. However, these results contradict an earlier publication by Wang et al. 31 who reported that m^6^A might be required for maintenance of the ground state. Contrary to the two more recent publications, their cells with knockdown of METTL3 and METTL14 were unable to maintain pluripotency and differentiated 31. However, considering the results by Batista et al. and Geula et al., the cells used by Wang et al. might have already been primed for differentiation. Lack of m^6^A would then allow differentiation‐specific transcripts to persist, which would lead to commitment to differentiation.

The observation that primed cells are more restricted in their differentiation has been speculated to correlate with hypermethylation, where chromatin is more condensed and DNA is less accessible 32. Naïve cells in both species have been shown to be hypomethylated. This is particularly evident in female cells, as both X chromosomes are still active in the early embryo, which is also reported in naïve pluripotent stem cells. X‐inactivation by heterochromatin formation is observed in primed cells and thus can be a marker to distinguish between both states 14, 15, 18, 20.

## Gene Editing Efficiency

The efficiency of homologous recombination is significantly higher in mESCs in comparison to hESCs 33. This has led to the hypothesis that the naïve state might be more amenable to gene editing. Buecker et al. 34 generated human naïve‐like cells by transgene expression and measured random insertion of 10–20 kb cassettes containing a fluorescent marker and drug resistance gene. They showed a 200‐fold increase of insertion frequency in their naïve‐like cells. The authors also targeted hypoxanthine‐guanine phosphoribosyltransferase with a puromycin selection cassette with 4–4.5 kb homology arms and reported correctly targeted insertion rates as high as 1% but did not compare this to primed cells 34.

A different approach was taken by Gafni et al. 15, who measured rates of correct insertion in the two endogenous loci *COL1A* and *OCT4* by using a puromycin selection cassette with homology arms of 2.1–2.5 kb and 4–4.5 kb in length, respectively. They showed relatively high correctly targeted integration rates of 11%–14.5% in naïve cells, whereas integration in primed cells was low (0%–0.3%) 15. Both groups show high rates of homologous recombination in naïve cells using standard electroporation techniques of dsDNA plasmid template and without the need for nucleases—this is an advantage as site‐specific nucleases including CRISPR‐cas9 have been shown to exhibit off‐target effects, which can only be ruled out after whole genome sequencing 35. A reason for the difference between primed and naïve editing efficiencies may be due to chromatin accessibility which has been shown to affect gene editing 36—the more open chromatin state in naïve cells might facilitate targeting. However, conclusive evidence for this is lacking. Moreover, gene editing in primed cells is technically challenging due to the requirement of clonal steps. Increased single cell survival of naïve cells, together with higher rates of proliferation, facilitates genetic manipulations that require cloning steps 34.

## Strategies of Derivation and the Pluripotency Network

**Figure 2:**
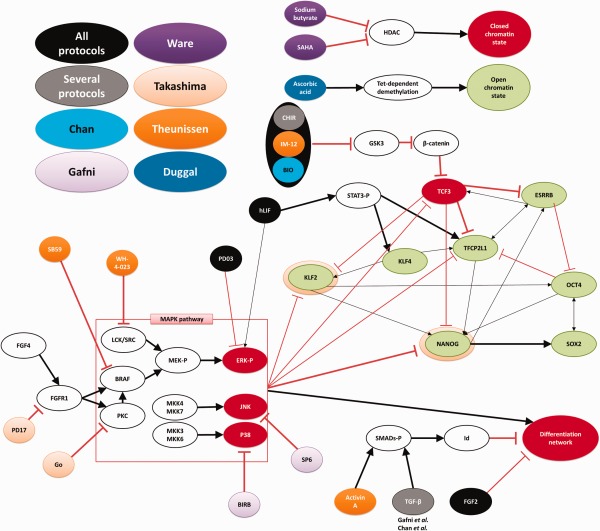
Effects of naïve conversion strategies on the core naïve pluripotency network. Components directly driving naivety are presented in green, whereas components with naïve repressive function are shown as red. Thick arrows indicate several publications have reported this interaction independently. External components present in conversion media of different protocols are color coded. All protocols require addition of a GSK3β inhibitor: The Theunissen et al. protocol uses IM‐12 19, the Chan et al. protocol BIO 17, whereas all other protocols use CHIR99021. KLF2 and NANOG are overexpressed in the protocol developed by Takashima et al. 14, which is depicted by a halo around these transcription factors. TGFβ is used as a supplement by Gafni et al. 15 and is present in the basal media of the protocol devised by Chan et al. 17. The PKC inhibitor Gö6983 is optional in the protocol by Gafni et al. 15. Abbreviations used: BIRB, BIRB796; CHIR, CHIR99021; FGF4, fibroblast growth factor; Go, Gö6983; GSK3, glycogen synthase kinase 3; HDAC, histone deacetylase; hLIF, human leukemia inhibitory factor; PD17, PD173074; PD03, PD0325901; SB59, SB590885; SP6, SP600125; TGFβ, transforming growth factor beta 14, 16, 17, 18, 19, 20, 32, 37, 38, 39.

Maintenance of the naïve state requires 2i and LIF, which stabilize the naïve pluripotency network (Supporting Information Fig. S1). This network of transcription regulators consists of several key elements including NANOG, OCT4, SOX2, and KLF2 (Supporting Information Fig. S2) 12, 32, 37, 38, 39, 40. Current strategies rely on reinforcing the naïve pluripotency network and repressing differentiation and negative influences (Fig. 2). Naïve‐like cells were initially reported by Buecker et al. 34, who ectopically expressed transcription factors OCT4, SOX2, KLF4, cMyc, and NANOG. However, maintenance of this state was only possible with constant transgene expression 34.

Takashima et al. 14 overexpressed KLF2 and NANOG which are key transcription regulators for the acquisition of the naïve state. This rewiring of the pluripotency circuitry, together with their media formulation, leads to stable self‐renewing naïve pluripotent cells even after silencing of transgene expression 14. Five other recent publications report achieving naïve pluripotency without using any transgenes, by using different media compositions containing small molecule inhibitors and growth factors (Supporting Information Table S1) 15, 17, 18, 19, 20. All available protocols rely on inhibition of MEK and GSK3β and on addition of bFGF, which represses differentiation. Most protocols also continuously add hLIF, with the exception of Ware et al. 20. Other components either cause demethylation, repress differentiation or are inhibitors targeting MAPK pathways (summarized in Fig. 2). Some teams have reported that low oxygen level aids conversion 14, 19, 20, whereas others have reported no benefit of lowered oxygen 15, 17, 18 (Supporting Information Table S1).

Different strategies (Supporting Information Fig. S3) have been used for naïve derivation, so the resulting cells have different properties. Evidence supporting naivety of these cells is summarized in Supporting Information Table S2. Chan et al. 17 were able to generate cells without transgene expression but did not bring forward as much evidence as most other protocols. Gafni et al. were able to demonstrate differentiation ability of their naïve cells by generating cross‐species chimeric mouse embryos containing differentiated cells derived from the human naïve cells in several different tissues. However, they did not perform in vitro differentiation 15. Ground state cells generated by Valamehr et al. 18 did not exhibit naïve morphology and their protocol requires single cell cloning, however this workflow has been designed for high throughput generation of homogenous cells which share properties of naivety, and therefore has its own applications. The protocol by Ware et al. requires reverse toggling with HDAC inhibitors and is not particularly efficient. However, Ware et al. 20 have generated a stable naïve cell line Elf1, which is banked and available. Takashima et al. bring forward comprehensive evidence for the naivety of their cells, including evidence for a switch to mitochondrial respiration. However, this protocol requires transgene delivery and therefore is less practical 14. Theunissen et al. 19 also show that their cells are naïve, however their protocol can induce karyotypic abnormalities and their naïve female cells undergo X inactivation, indicating a later stage in development. The most recent publication (Duggal et al.) includes a demonstration of enhanced directed differentiation in comparison to their primed parental cells 16. Reproduction of these protocols by other laboratories will establish how robust they are.

## Conclusions

The concept of naïve hPSCs has been contentious. Pera 41 argues that since this state was actively searched for in humans, it is highly likely that it is purely an artifact generated in the lab. However, Wang et al. used RNA‐seq data which was available from cells taken directly from the ICM of early embryos and showed a tight correlation to naïve cells generated in vitro 27.

This was confirmed when Huang, Maruyama, and Fan took a systems biology approach and compared datasets from many previous publications 42. Their analysis revealed poor conservation of gene networks between mPSCs and hPSCs but a high resemblance to the ICM of their respective blastocysts. They also found variations in transcriptomes from different naïve conversion protocols, but all established naïve cells showed clear resemblance to human late preimplantation embryos. According to this study, naïve cells generated by Takashima et al. 14 and Theunissen et al. 19 most closely resembled the human preimplantation blastocyst. The protocols by Valamehr et al. 18 and Duggal et al. 16 were not included in the study. In conclusion, the authors propose comparing the combination of transcriptome analysis and epigenetic characterization to in vivo data from embryogenesis as a gold standard for naivety 42.

The description of just two states, naïve and primed, is an oversimplification 11, 27, 43, 44. Two studies 27, 43 used single‐cell RNA‐seq and reported a polyclonal spectrum of cell states ranging between these extremes and that naïve PSCs are present as a subpopulation in cultures previously considered entirely primed. Wang et al. 27 used a reporter system based on the endogenous retrovirus HERVH's LTR7 promoter which is only active in naïve cells. This approach showed a consistent 4% of cells with naïve reporter expression which can be selected for using 2i and LIF and do not need prior conversion. Recently, Wu et al. were able to capture another alternative state designated “region‐selective primed” pluripotency in vitro in both mouse and human which are distinct from both naïve and primed states 44.

There remain many challenges in the field of naïve pluripotency. All protocols for generating human naïve PSCs yield slightly different cellular states. It is still unclear which of these is closest to its in vivo counterpart. The in vivo naïve state is inherently transient, so continuous in vitro culture may be detrimental. For example, female cells maintained in the naïve state that do not exhibit X‐inactivation might suffer from double dosage effects. With protocols now readily available which allow the generation and maintenance of naïve cells, these questions can be addressed. Meanwhile, their faster rate of growth, single cell survival, and enhanced gene editing efficiency will be used. In the near future, naïve hPSCs may be useful for accessing paths of differentiation which have been previously unreachable.

## Author Contributions

B.T.D.: conception and design, collection and/or assembly of data, and manuscript writing; R.F.: conception and design, manuscript writing, and financial support; S.A.C.: conception and design, manuscript writing, financial support, administrative support, and final approval of manuscript.

## Disclosure of Potential Conflicts of Interest

Research funding includes grants from BBSRC industrial case DPhil training grant BB/L015447/1 with industrial partner F. Hoffmann‐La Roche AG (B.T.D.) and EU IMI STEMBANCC grant number 115439 (R.F., S.A.C.).

## Supporting information

Supplementary Information Figure 1

Supplementary Information Figure 2

Supplementary Information Figure 3

Supplementary Information Table S1

Supplementary Information Table S2

Supplementary Information Figure and Table Legends

## References

[1] Evans MJ , Kaufman MH . Establishment in culture of pluripotential cells from mouse embryos. Nature 1981;292:154–156. 7242681 10.1038/292154a0

[2] Brook FA , Gardner RL . The origin and efficient derivation of embryonic stem cells in the mouse. Proc Natl Acad Sci 1997;94:5709–5712. 10.1073/pnas.94.11.5709 PMC20843 9159137

[3] Ying Q‐L , Wray J , Nichols J et al. The ground state of embryonic stem cell self‐renewal. Nature 2008;453:U519–U515. 10.1038/nature06968 PMC5328678 18497825

[4] Bradley A , Evans M , Kaufman MH et al. Formation of germ‐line chimaeras from embryo‐derived teratocarcinoma cell lines. Nature 1984;309:255–256. 6717601 10.1038/309255a0

[5] Tesar PJ , Chenoweth JG , Brook FA et al. New cell lines from mouse epiblast share defining features with human embryonic stem cells. Nature 2007;448:U196–U110. 10.1038/nature05972 17597760

[6] Brons IGM , Smithers LE , Trotter MWB et al. Derivation of pluripotent epiblast stem cells from mammalian embryos. Nature 2007;448:191–195. 17597762 10.1038/nature05950

[7] Ozawa M , Kawakami E , Sakamoto R et al. Development of FGF2‐dependent pluripotent stem cells showing naive state characteristics from murine preimplantation inner cell mass. Stem Cell Res 2014;13:75–87. 24835670 10.1016/j.scr.2014.04.012

[8] Nichols J , Smith A . Naive and primed pluripotent states. Cell Stem Cell 2009;4:487–492. 19497275 10.1016/j.stem.2009.05.015

[9] Zhou H , Li W , Zhu S et al. Conversion of mouse epiblast stem cells to an earlier pluripotency state by small molecules. J Biol Chem 2010;285:29676–29680. 20705612 10.1074/jbc.C110.150599 PMC2943300

[10] Fang R , Liu K , Zhao Y et al. Generation of naive induced pluripotent stem cells from rhesus monkey fibroblasts. Cell Stem Cell 2014;15:488–496. 25280221 10.1016/j.stem.2014.09.004

[11] Mascetti VL , Pedersen RA . Naivete of the human pluripotent stem cell. Nat Biotechnol 2014;32:68–70. 24406934 10.1038/nbt.2789

[12] Buecker C , Srinivasan R , Wu Z et al. Reorganization of enhancer patterns in transition from naive to primed pluripotency. Cell Stem Cell 2014;14:838–853. 24905168 10.1016/j.stem.2014.04.003 PMC4491504

[13] Hanna J , Cheng AW , Saha K et al. Human embryonic stem cells with biological and epigenetic characteristics similar to those of mouse ESCs. Proc Natl Acad Sci USA 2010;107:9222–9227. 10.1073/pnas.1004584107 PMC2889088 20442331

[14] Takashima Y , Guo G , Loos R et al. Resetting transcription factor control circuitry toward ground‐state pluripotency in human. Cell 2014;158:1254–1269. 25215486 10.1016/j.cell.2014.08.029 PMC4162745

[15] Gafni O , Weinberger L , Mansour AA et al. Derivation of novel human ground state naive pluripotent stem cells. Nature 2013;504:282–286. 24172903 10.1038/nature12745

[16] Duggal G , Warrier S , Ghimire S et al. Alternative Routes to Induce Naive Pluripotency in Human Embryonic Stem Cells. Stem Cells. 2015. 10.1002/stem.2071 26108678

[17] Chan Y‐S , Goeke J , Ng J‐H et al. Induction of a human pluripotent state with distinct regulatory circuitry that resembles preimplantation epiblast. Cell Stem Cell 2013;13:663–675. 24315441 10.1016/j.stem.2013.11.015

[18] Valamehr B , Robinson M , Abujarour R et al. Platform for induction and maintenance of transgene‐free hiPSCs resembling ground state pluripotent stem cells. Stem Cell Rep 2014;2:366–381. 10.1016/j.stemcr.2014.01.014 PMC3964282 24672758

[19] Theunissen TW , Powell BE , Wa H et al. Systematic identification of culture conditions for induction and maintenance of naive human pluripotency. Cell Stem Cell 2014;15:471–487. 25090446 10.1016/j.stem.2014.07.002 PMC4184977

[20] Ware CB , Nelson AM , Mecham B et al. Derivation of naive human embryonic stem cells. Proc Natl Acad Sci USA 2014;111:4484–4489. 10.1073/pnas.1319738111 PMC3970494 24623855

[21] Brinster R , Troike D . Requirements for blastocyst development in vitro. J Anim Sci 1978;49:26–34. 45481 10.1093/ansci/49.supplement_ii.26

[22] Martin KL , Leese HJ . Role of glucose in mouse preimplantation embryo development. Mol Reprod Dev 1995;40:436–443. 7598909 10.1002/mrd.1080400407

[23] Zhou W , Choi M , Margineantu D et al. HIF1α induced switch from bivalent to exclusively glycolytic metabolism during ESC‐to‐EpiSC/hESC transition. EMBO J 2012;31:2103–2116. 22446391 10.1038/emboj.2012.71 PMC3343469

[24] Teslaa T , Teitell MA . Pluripotent stem cell energy metabolism: An update. EMBO J 2015;34:138–153. 25476451 10.15252/embj.201490446 PMC4337063

[25] Van Bortle K , Corces VG . Lost in transition: Dynamic enhancer organization across naive and primed stem cell states. Cell Stem Cell 2014;14:693–694. 24905156 10.1016/j.stem.2014.05.004 PMC4115280

[26] Factor DC , Corradin O , Zentner GE et al. Epigenomic comparison reveals activation of “seed” enhancers during transition from naive to primed pluripotency. Cell Stem Cell 2014;14:854–863. 24905169 10.1016/j.stem.2014.05.005 PMC4149284

[27] Wang J , Xie G , Singh M et al. Primate‐specific endogenous retrovirus‐driven transcription defines naive‐like stem cells. Nature 2014;516:405–409. 25317556 10.1038/nature13804

[28] Grow E , Flynn R , Chavez S et al. Intrinsic retroviral reactivation in human preimplantation embryos and pluripotent cells. Nature 2015;522:221–225. 25896322 10.1038/nature14308 PMC4503379

[29] Batista Pedro J , Molinie B , Wang J et al. m6A RNA modification controls cell fate transition in mammalian embryonic stem cells. Cell Stem Cell 2014;15:707–719. 25456834 10.1016/j.stem.2014.09.019 PMC4278749

[30] Geula S , Moshitch‐Moshkovitz S , Dominissini D et al. Stem cells. m6A mRNA methylation facilitates resolution of naive pluripotency toward differentiation. Science 2015;347:1002–1006. 25569111 10.1126/science.1261417

[31] Wang Y , Li Y , Toth JI et al. N6‐methyladenosine modification destabilizes developmental regulators in embryonic stem cells. Nat Cell Biol 2014;16:191–198. 24394384 10.1038/ncb2902 PMC4640932

[32] Ye S , Liu D , Ying Q‐L . Signaling pathways in induced naïve pluripotency. Curr Opin Genet Dev 2014;28:10–15. 25173148 10.1016/j.gde.2014.08.002 PMC4262619

[33] Zwaka TP , Thomson JA . Homologous recombination in human embryonic stem cells. Nat Biotechnol 2003;21:319–321. 12577066 10.1038/nbt788

[34] Buecker C , Chen H‐H , Polo JM et al. A murine ESC‐like state facilitates transgenesis and homologous recombination in human pluripotent stem cells. Cell Stem Cell 2010;6:535–546. 20569691 10.1016/j.stem.2010.05.003 PMC3162213

[35] Fu Y , Foden JA , Khayter C et al. High‐frequency off‐target mutagenesis induced by CRISPR‐Cas nucleases in human cells. Nat Biotechnol 2013;31:822–826. 23792628 10.1038/nbt.2623 PMC3773023

[36] Wu X , Scott DA , Kriz AJ et al. Genome‐wide binding of the CRISPR endonuclease Cas9 in mammalian cells. Nat Biotechnol 2014;32:670–676. 24752079 10.1038/nbt.2889 PMC4145672

[37] Dunn SJ , Martello G , Yordanov B et al. Defining an essential transcription factor program for naive pluripotency. Science 2014;344:1156–1160. 24904165 10.1126/science.1248882 PMC4257066

[38] Hackett JA , Surani MA . Regulatory principles of pluripotency: From the ground state up. Cell Stem Cell 2014;15:416–430. 25280218 10.1016/j.stem.2014.09.015

[39] Niwa H . The pluripotency transcription factor network at work in reprogramming. Curr Opin Genet Dev 2014;28:25–31. 25173150 10.1016/j.gde.2014.08.004

[40] Yang S‐H , Kalkan T , Morissroe C et al. Otx2 and Oct4 drive early enhancer activation during embryonic stem cell transition from naive pluripotency. Cell Rep 2014;7:1968–1981. 24931607 10.1016/j.celrep.2014.05.037 PMC4074343

[41] Pera MF . In search of naivety. Cell Stem Cell 2014;15:543–545. 25517463 10.1016/j.stem.2014.10.013

[42] Huang K , Maruyama T , Fan G . The naive state of human pluripotent stem cells: A synthesis of stem cell and preimplantation embryo transcriptome analyses. Cell Stem Cell 2014;15:410–415. 25280217 10.1016/j.stem.2014.09.014 PMC5507179

[43] Hough SR , Thornton M , Mason E et al. Single‐cell gene expression profiles define self‐renewing, pluripotent, and lineage primed states of human pluripotent stem cells. Stem Cell Rep 2014;2:881–895. 10.1016/j.stemcr.2014.04.014 PMC4050352 24936473

[44] Wu J , Okamura D , Li M et al. An alternative pluripotent state confers interspecies chimaeric competency. Nature 2015;521:316–321. 25945737 10.1038/nature14413 PMC5278765

