# Supplementary figures and images for: The Current State of Naïve Human Pluripotency
Source: Stem Cells. 2015 Jul 14;33(11):3181–6. doi: 10.1002/stem.2085 (PMC4833179; doi:10.1002/stem.2085)

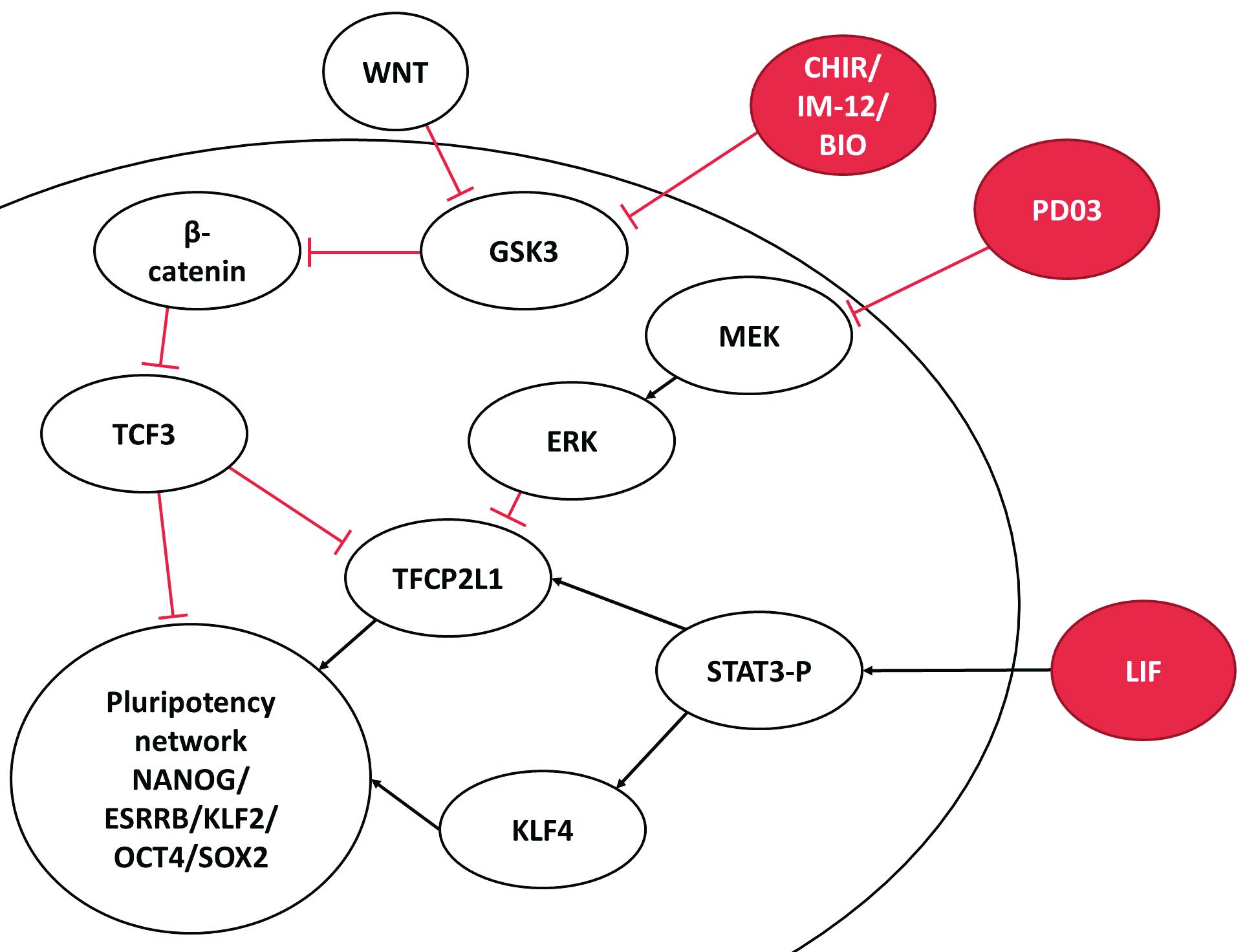

Supplement: Supplementary file 1 — Supplementary Information Figure 1 [file STEM-33-3181-s001.tif]

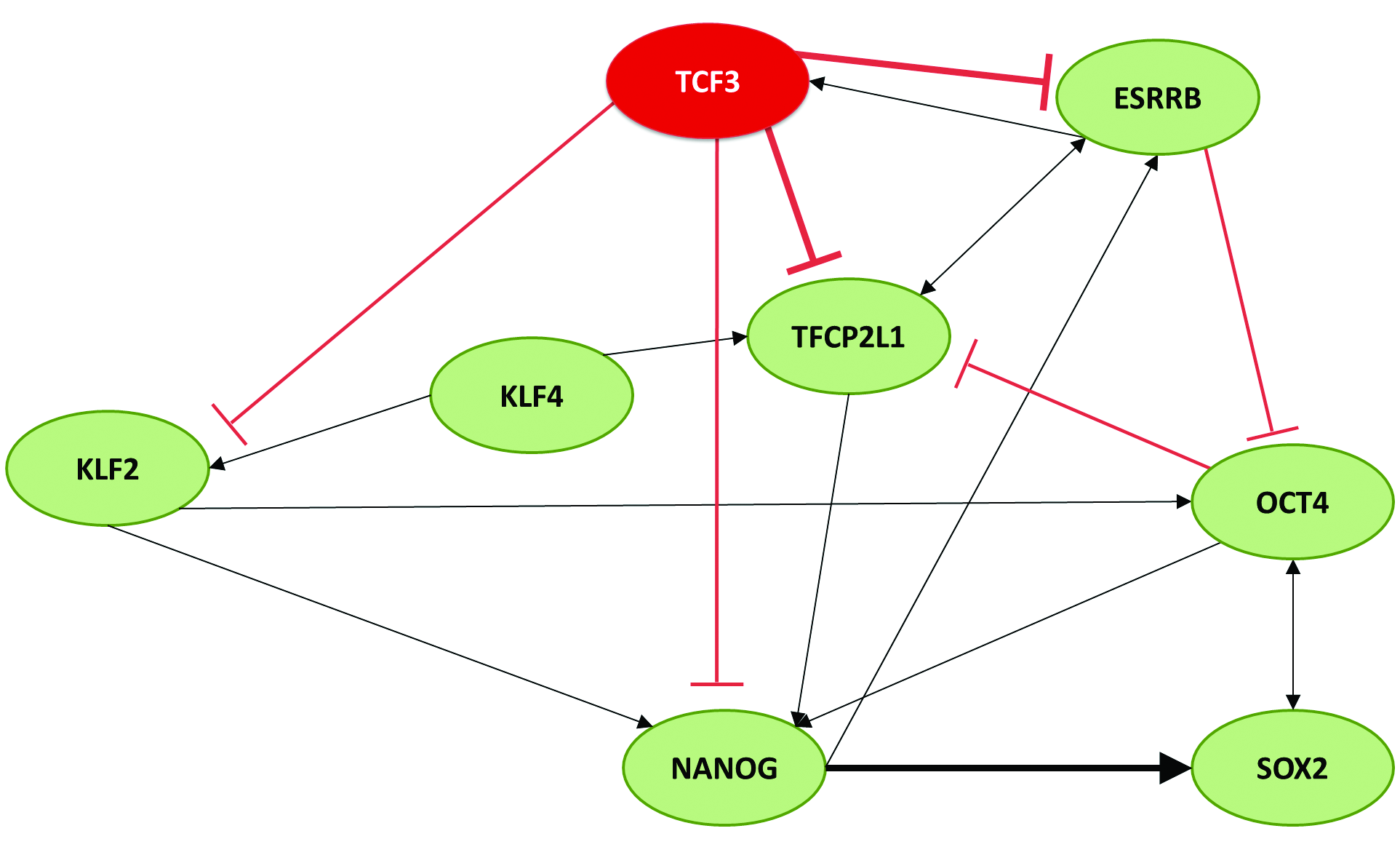

Supplement: Supplementary file 2 — Supplementary Information Figure 2 [file STEM-33-3181-s002.tif]

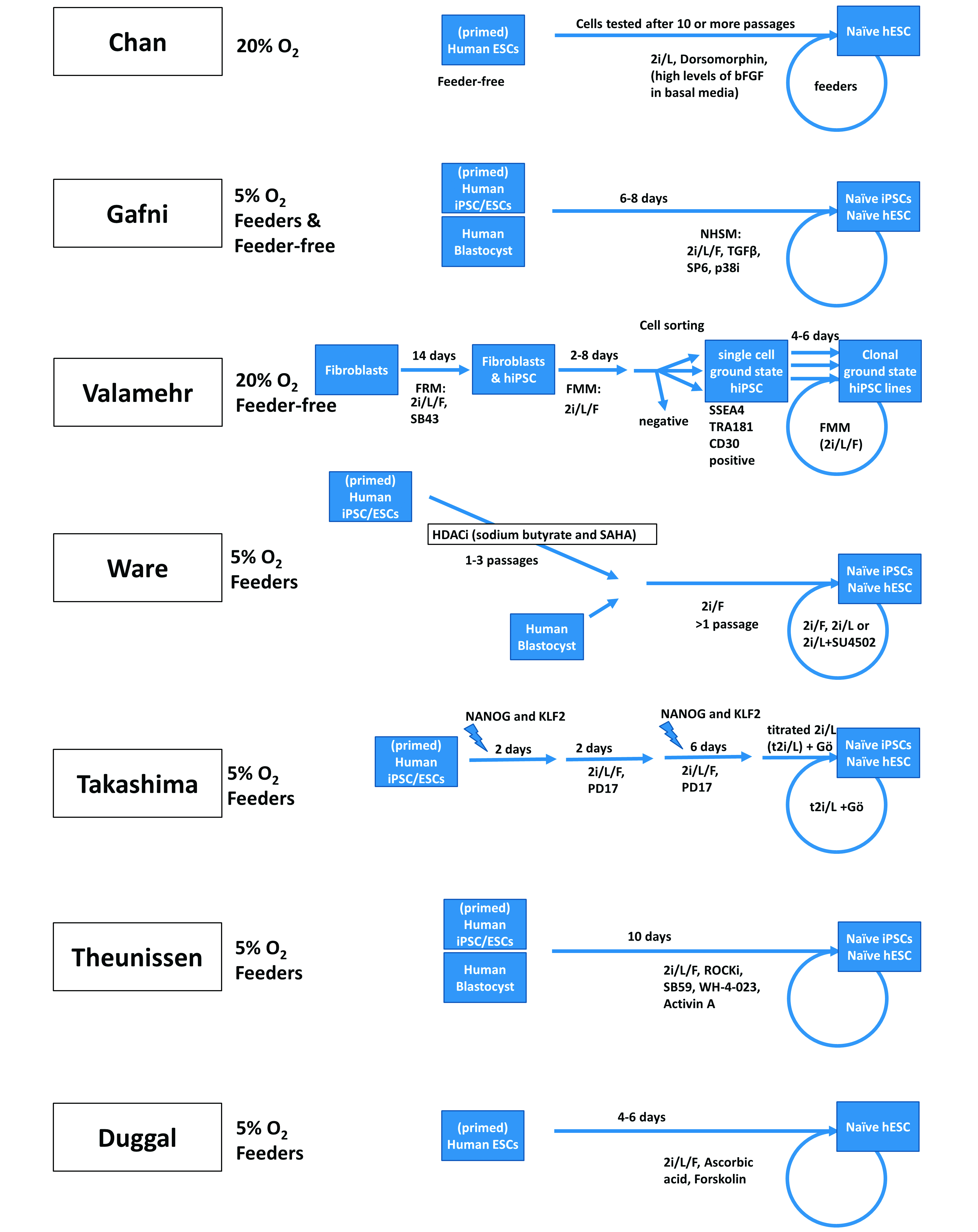

Supplement: Supplementary file 3 — Supplementary Information Figure 3 [file STEM-33-3181-s003.tif]
