# Supplementary material for: The Current State of Naïve Human Pluripotency
Source: Stem Cells. 2015 Jul 14;33(11):3181–6. doi: 10.1002/stem.2085 (PMC4833179; doi:10.1002/stem.2085)
Supplement: Supplementary file 4 — Supplementary Information Table S1 [file STEM-33-3181-s004.doc]

|  | **Component** | **Function** | **Chan *et al.*** | **Gafni *et al.*** | **Valamehr *et al.*** | **Ware *et al.*** | **Takashima *et al.*** | **Theunissen *et al.*** | **Duggal *et al.*** |
| --- | --- | --- | --- | --- | --- | --- | --- | --- | --- |
| **Small molecules** | PD0325901 | MEKi | C/M | C/M | C/M | C/M | C/M | C/M | C/M |
|  | CHIR-99021 | GSK-3βi | C/M | C/M | C/M | C/M | C/M | C/M | C/M |
|  | BlO |  |  |  |  |  |  |  |  |
|  | IM-12 |  |  |  |  |  |  |  |  |
|  | Thiazovivin/Y-27632 | ROCKi | initially | (C/M) | C/M |  |  | C/M |  |
|  | Go6983 | PKCi |  | (C/M) |  |  | C |  |  |
|  | Ascorbic acid | Tet-dependant demethylation |  |  |  |  |  |  | C/M |
|  | BIRB/SB203580/SB202190 | p38i |  | C/M |  |  |  |  |  |
|  | Dorsomorphin | BMPi | C/M |  |  |  |  |  |  |
|  | Forskolin | stimulates cAMP production |  |  |  |  |  |  | C/M |
|  | PD173074 | FGFR1i |  |  |  |  | C |  |  |
|  | SAHA | HDACi |  |  |  | preculture |  |  |  |
|  | SB431542 | ALKi |  |  | C |  |  |  |  |
|  | SB590885 | BRAFi |  |  |  |  |  | C/M |  |
|  | Sodium butyrate | HDACi |  |  |  | preculture |  |  |  |
|  | SP600125 | JNKi, MAPKi |  | C/M |  |  |  |  |  |
|  | WH-4-023 | LCKC/SRCi inhibitor |  |  |  |  |  | C/M |  |
| **Growth factors and signalling peptides** | bFGF/FGF2 | Keeps cells undifferentiated | * | C/M | C/M | C/M | C | C/(M) | C/M |
|  | hLIF | Activates JAK/STAT and MAPK cascades, reduces differentiation | C/M | C/M | C/M | C | C/M | C/M | C/M |
|  | Activin A | Activation of SMAD2/3 |  |  |  |  |  | C/M |  |
|  | Insulin | Metabolic and catabolic regulator |  | C/M |  |  |  |  |  |
|  | TGFb | Activation of SMAD2/3 | * | C/M |  |  |  |  |  |
| **Growth conditions** | Feeder-free |  | C | C/M | C/M |  |  |  | (M) |
|  | Feeders |  | M |  |  | C/M | C/M | C/M | C/M |
|  | Oxygen levels |  | 20% | 20% | 20% | 5% | 5% | 5% | 5% |
|  | Basal media |  | TeSR1 | KO DMEM, AlbuMAX1, NEAA, N2 | DMEM/F12, KSR, NEAA, Glutamine, N2, B27. (No N2 or B27 for maintenance) | DMEM/F12, KSR, NEAA, Glutamax, sodium pyruvate | DMEM/F-12, KSR, NEAA, Glutamine. Maintenance in N2B27 medium | DMEM/F12, Neurobasal, BSA, NEAA, Glutamine, N2, B27 | KO-DMEM, KSR, NEAA, Glutamine |

Table S1
