# Supplementary material for: The Current State of Naïve Human Pluripotency
Source: Stem Cells. 2015 Jul 14;33(11):3181–6. doi: 10.1002/stem.2085 (PMC4833179; doi:10.1002/stem.2085)
Supplement: Supplementary file 5 — Supplementary Information Table S2 [file STEM-33-3181-s005.doc]

Table S2

|  |  | transgene-free | normal karyotype | mES morphology | no cell sorting required | good efficiency | naïve specific RNA profile | | |
| --- | --- | --- | --- | --- | --- | --- | --- | --- | --- |
|  |  |  |  |  |  |  | RNA-seq | array | qPCR |
| Chan *et al.* | 2013 | ✓ | ✓ | ✓ | ✓ | ✓ | ✓ | ? | ✓ |
| Gafni *et al.* | 2013 | ✓ | ✓ | ✓ | ✓ | ✓ | ? | ✓ | ✓ |
| Valamehr *et al.* | 2014 | ✓ | ✓ | X | X | ✓ | ? | ✓ | ? |
| Ware *et al.* | 2014 | ✓ | ✓ | ✓ | ✓ | X | ? | ? | ? |
| Takashima *et al.* | 2014 | X | ✓ | ✓ | ✓ | ✓ | ✓ | ✓ | ? |
| Theunissen *et al.* | 2014 | ✓ | X | ✓ | ✓ | ✓ | ? | ✓ | ✓ |
| Duggal *et al.* | 2015 | ✓ | ✓ | ✓ | ✓ | ✓ | ? | ✓ | ✓ |

|  |  | in vitro differentiation | | | teratoma formation into all 3 germ layers | XaXa | Hypo-methylated | oxidative phosphorlyation | media used to isolate mESC |
| --- | --- | --- | --- | --- | --- | --- | --- | --- | --- |
|  |  | spontaneous (EB) | directed | compared to primed |
| Chan *et al.* | 2013 | ✓ | ? | ? | ✓ | ? | ? | ? | ? |
| Gafni *et al.* | 2013 | ✓ | ? | ? | ✓ | ✓ | ✓ | ? | ? |
| Valamehr *et al.* | 2014 | ✓ | ✓ | ? | ✓ | a | ✓ | ? | ? |
| Ware *et al.* | 2014 | ? | ? | ? | ✓ | ✓ | ✓ | b | ? |
| Takashima *et al.* | 2014 | ✓ | ✓ | ? | ✓ | ✓ | ✓ | ✓ | ? |
| Theunissen *et al.* | 2014 | ? | ✓ | ? | ✓ | X | ? | ? | ? |
| Duggal *et al.* | 2015 | ✓ | ✓ | ✓ | ? | ✓ | ✓ | ? | ✓ |
