# Supplementary material for: The Current State of Naïve Human Pluripotency
Source: Stem Cells. 2015 Jul 14;33(11):3181–6. doi: 10.1002/stem.2085 (PMC4833179; doi:10.1002/stem.2085)
Supplement: Supplementary file 6 — Supplementary Information Figure and Table Legends [file STEM-33-3181-s006.docx]

# Supplementary Table and Figure legends

Figure S1: **The effect of 2i+LIF on the pluripotency network.** Simplified schematic showing the effect of the GSK3β inhibitor CHIR99021, the MEK/ERK pathway inhibitor PD0325901 (in combination known as 2i) and LIF. Both inhibitors suppress negative cues, whereas LIF promotes the pluripotency network by activation of STAT3. [14, 16-20, 32, 37-39]

Figure S2: **The core naive pluripotency transcription factor** **network.** Factors driving naivety are presented in green, whereas factors with repressive function are shown as red. Thick arrows indicate a high confidence interaction, meaning several publications have reported this independently. [14, 16-20, 32, 37-39]

Figure S3: **Strategies employed by different naïve conversion protocols.** Where nothing is specified for maintenance, there is no change in culture conditions following derivation. The protocol developed by Takashima *et al.* [14] requires two transient transfections of *Nanog* and *Klf2*, whereas the remaining protocols are transgene-free. Abbreviations used: CHIR99021 + PD0325901 (collectively 2i), 2i + hLIF + FGF2 (2i/L/F), naïve human stem cell medium (NHSM), SP6 (SP600125), BIRB796 or SB203580 or SB202190 (p38i), Fate Reprogramming Medium (FRM), SB431542 (SB43), Fate Maintenance Medium (FMM), histone deacetylase (HDAC), suberoylanilide hydroxamic acid (SAHA), PD173074 (PD17), Gö6983 (Gö), SB590885 (SB59). [14-20]

Table S1: **Summary of different media compositions used for conversion and subsequent maintenance of the naïve state.** “C” indicates a requirement for conversion, whereas the “M” shows a requirement for maintenance of the naïve state. Brackets mean that the addition is optional and the asterisk (*) indicates high levels present in the basal media. [14-20]

Table S2: **Summary of evidence for naivety for cells generated using different protocols.** Question marks indicate that this test or assay was not performed. “a” indicates high X chromosome gene expression but low Xist transcript levels. “b” indicates mitochondrial morphological assessment only. [14-20]
